# Altered vector competence in an experimental mosquito-mouse transmission model of Zika infection

**DOI:** 10.1371/journal.pntd.0006350

**Published:** 2018-03-05

**Authors:** Ryuta Uraki, Andrew K. Hastings, Andrea Gloria-Soria, Jeffrey R. Powell, Erol Fikrig

**Affiliations:** 1 Section of Infectious Diseases, Department of Internal Medicine, Yale University School of Medicine, New Haven, Connecticut, United States of America; 2 Department of Ecology and Evolutionary Biology, Yale University, New Haven, Connecticut, United States of America; 3 Howard Hughes Medical Institute, Chevy Chase, Maryland, United States of America; Fundaçao Oswaldo Cruz, BRAZIL

## Abstract

Few animal models of Zika virus (ZIKV) infection have incorporated arthropod-borne transmission. Here, we establish an *Aedes aegypti* mosquito model of ZIKV infection of mice, and demonstrate altered vector competency among three strains, (Orlando, ORL, Ho Chi Minh, HCM, and Patilas, PAT). All strains acquired ZIKV in their midguts after a blood meal from infected mice, but ZIKV transmission only occurred in mice fed upon by HCM, and to a lesser extent PAT, but not ORL, mosquitoes. This defect in transmission from ORL or PAT mosquitoes was overcome by intrathoracic injection of ZIKV into mosquito. Genetic analysis revealed significant diversity among these strains, suggesting a genetic basis for differences in ability for mosquito strains to transmit ZIKV. The intrathoracic injection mosquito-mouse transmission model is critical to understanding the influence of mosquitoes on ZIKV transmission, infectivity and pathogenesis in the vertebrate host, and represents a natural transmission route for testing vaccines and therapeutics.

## Author summary

Zika virus (ZIKV), an emerging flavivirus, is associated with severe clinical outcomes, including Guillain-Barre syndrome and birth defects. Transmission of ZIKV is primarily mosquito-borne, but the complete transmission cycle from mammalian host to mosquito and back to mammalian host has not yet been demonstrated experimentally. Here, for the first time, we establish a transmission model of ZIKV between mice lacking both interferon α and interferon γ receptor (AG129) and the *Aedes aegypti* mosquito, the main vector of ZIKV. We also highlight differences in vector competence among a common laboratory strain, Orlando (ORL), and two wild-caught strains, Ho Chi Minh (HCM) and Patillas (PAT), of the *Aedes aegypti* mosquito using oral feeding on infected mice and intrathoracic inoculation of ZIKV. Our results suggest different ZIKV susceptibility among these strains, which could be relevant as this virus spreads throughout the globe. In addition, the mosquito transmission model demonstrated here can be used to better understand the effect mosquitoes have on replication and pathogenesis of this virus.

## Introduction

Zika virus (ZIKV), a member of the *flaviviridae* family, was originally isolated from a sentinel monkey in the Zika forest of Uganda in 1947 [1]. The first case of ZIKV infection in humans was reported in Nigeria in 1954 [2]. For half a century, serologic evidence suggests that the virus circulated in Africa and Southeast Asia, although fewer than 20 symptomatic infections were documented [3]. Recently, a large epidemic in South and Central America and the Caribbean, which also spread to limited areas in the southernmost parts of the contiguous United States, has affected over a million people [4]. Historically, ZIKV has manifested as a relatively mild self-limiting illness, with fever, rash, malaise and headache reported as the most commons symptoms, and up to 80% of infected individuals remaining asymptomatic [3, 5, 6]. These new epidemics, however, have been associated with Guillain-Barre syndrome in adults, and congenital deformities and neurologic syndromes in newborns [7–9]. The increase in disease severity and rapid spread of ZIKV has led to increasing alarm across the globe.

ZIKV is thought to spread mainly through the bite of an infected mosquito, similar to other flaviviruses such as dengue and West Nile virus [10]. The major mosquito vector for ZIKV is *Aedes aegypti*, which most likely originated in Africa and is now endemic in tropical and subtropical locations. While ZIKV has regularly been found to persist in populations of *Ae*. *aegypti* in areas where this virus is circulating [8], data related to the experimental transmission of *Ae*. *aegypti* with ZIKV in a laboratory setting has been sparse [11–13]. A recent publication has shown experimental transmission from mosquito to mouse and back to mosquito [14], but, to date, no experimental model of the transmission cycle from infected mammalian host to mosquito and back to mammalian host has been demonstrated. Also, the importance of a robust model for mosquito transmission of ZIKV is highlighted by recent work in a macaque model, showing that mosquito bite infection of animals significantly changes the viral replication dynamics and tissue tropism as compared to needle inoculation [15]. These studies were carried out with a contemporary pandemic strain of ZIKV, but additional work is planned to determine the significance of pre- and post-pandemic viral strains on replication within the mosquito vector. A significant amount of genetic diversity exists within the *Ae*. *aegypti* species [16], so we hypothesized that the genetic background of individual strains of *Ae*. *aegypti* plays an important role in vector competence for ZIKV. This hypothesis is supported by research showing the dissemination of ZIKV within the mosquito host differs among mosquito strains, from Dominican Republic, Brazil and U.S.A [17, 18], and species [17, 18] in the wild. ZIKV poses a significant public health risk, and exploring the relationship between vector and pathogen is an important factor in determining the risk imposed by individual populations of mosquitoes. It is also vital to explore the role of the vector in ZIKV replication, infectivity, and pathogenesis within the mammalian host. In this study, we demonstrate a transmission cycle between infected mice to mosquitoes and back to mice using three *Ae*. *aegypti* colonies, which were selected based on their genetic distinctness among tested strains, the Orlando (ORL) strain, a common laboratory colony, the Ho Chi Minh (HCM) strain and the Patilas (PAT) strain, both recently established colonies from the field (~14 generations and ~3 generations, respectively), and using mice lacking receptors for interferon α/β/γ (AG129 mice), which are susceptible to low levels of ZIKV [19]. We also show that intrathoracic inoculation with ZIKV results in highly infectious mosquitoes regardless of strain, which will be important for future studies on the effect of mosquitoes in ZIKV infection and represents a natural route of infection for future studies of vaccines and therapeutics.

## Materials and methods

### Ethics statement

All experiments were performed in accordance with guidelines from the Guide for the Care and Use of Laboratory Animals of the NIH. Protocols were reviewed and approved by the IACUC at Yale University School of Medicine (Assurance number A3230-01). Every effort was made to reduce distress in animals. Animals were anesthetized with ketamine/xylazine for mosquito infection experiments, and sacrificed using CO_2_ inhalation as recommended by the Yale IACUC.

### Viruses and cell lines

Vero cells (ATCC) were maintained in DMEM containing 10% FBS and antibiotics at 37°C with 5% CO_2_ and have been routinely confirmed to be mycoplasma free. *Aedes albopictus* C6/36 cells were grown in DMEM supplemented with 10% FBS, 1% tryptose phosphate, and antibiotics at 30°C with 5% CO_2_. Mexican strain ZIKV (ZIKV^MEX^, Accession number KX446950), MEX2-81, was obtained from the University of Texas Medical Branch at Galveston’s World Reference Center for Emerging Viruses and Arboviruses and propagated in C6/36 insect cells.

### Mosquito strains

Eggs from ORL and HCM strains of *Ae*. *aegypti* were obtained from the Connecticut Agricultural Experimental Station and the HCM colony maintained at the Powell laboratory at Yale, respectively. Other laboratory strains arrived as eggs from different laboratories: Liverpool (D. Severson, University of Notre Dame), Rockefeller (G. Dimopoulos, Johns Hopkins School of Public Health). Field strains came as eggs collected from traps in the wild from Amacuzac, (Mexico) and Patillas (Puerto Rico)

### Mosquito care

Eggs were hatched in a shallow dish with distilled water with 2 parts brewer’s yeast (Bioserv #1710) and 3 parts desiccated liver powder (Bioserv #1320). After pupae emerged, mosquitoes were collected and placed in a small crystal dish with distilled water inside a 12” x 12” x 12” metal mesh cage (BioQuip #1450B). Adult mosquitoes are maintained on 10% sucrose feeders in walk-in incubator at 28^o^ C and ~80% humidity. Egg masses are generated via blood meal on naïve mice.

### Mouse models

Three to six week old *Ifnαr1*^*-/-*^*Ifnγr*^*-/-*^ mice (AG129 –SV129 background) were analyzed in this study. Approximately equal numbers of male and female mice were used. Mice were bred in a specific-pathogen-free facility at Yale University.

### Detection of virus-specific antibodies

Virus-specific antibodies (IgG) in serum from mice infected with ZIKV were analyzed by using an enzyme-linked immunosorbent assay (ELISA). Recombinant ZIKV Envelope protein (Mybiosource, MBS140822) (0.1μg/well) were coated to 96 well plates overnight at 4°C. After the plates were blocked with 2% skim milk for 1 h at room temperature, the plates were then incubated with serum samples serially diluted in PBS for 1 h at room temperature. After being washed with PBS-T three times, the plates were incubated with an HRP-conjugated anti-mouse IgG antibody. After the plates were washed again with PBS-T, 3,3’,5,5’-Tetramethylbenzidine solution was added to each well and incubated for 15 min at room temperature. The reaction was stopped by the addition of 1M H_2_SO_4_. The optical density at 450 nm (OD_450_) was measured and analyzed. The cut-off value was calculated as the mean OD_450_ + 3SD acquired from corresponding samples from three naïve mice.

### DNA extraction and microsatellite genotyping

Adult mosquitoes were flash frozen and total nucleic acids extracted from individual *Ae*. *aegypti* mosquitoes using the DNeasy Blood and Tissue kit (Qiagen) according to manufacturer instructions, with an additional RNAse A (Qiagen) step. Individual mosquitoes were genotyped as described in [20]. The microsatellite loci analyzed were: A1, B2, B3, A9 (tri-nucleotide repeats), and AC2, CT2, AG2, AC4, AC1, AC5, AG1, and AG4 (di-nucleotide repeats) [21]. Polymerase chain reactions were conducted as 10μl reactions using the Type-it Microsatellite PCR Master Mix (Qiagen), 25 nM of each forward primer, 250 nM of each reverse primer, and 500 nM of a fluorescently labeled M13 primer to allow multiplexing [22]. Microsatellite primer sequences, multiplex pairings and fluorescent primers are as described in [22]. PCR products were run for fragment analysis on an Applied Biosystems 3730xl DNA Genetic Analyzer with a GS 500 Rox internal size standard (Applied Biosystems) at the DNA Analysis Facility at Science Hill at Yale University. Microsatellite alleles were scored using GeneMapper v4.0 (Applied Biosystems).

### Infection of mosquitoes

For oral-infection, naïve AG129 mice are first infected subcutaneously with 10^5^ PFU of ZIKV^MEX^. One day before oral-infection, mosquitoes are collected via vacuum aspiration and placed in paper cups with mesh lids without sucrose. At 4 days post ZIKV injection, infected mice are anesthetized using Ketamine-Xylazine and laid on top of mesh lids to allow mosquitoes to take infectious blood meal. Fed mosquitoes are placed back in paper cups with mesh lids and maintained in triple containment for 7 or 10 days. For ZIKV injection experiments, mosquitoes were knocked-down on ice before transfer to a cold plate under a dissecting microscope. A pulled microcapillary needle was filled with ZIKV using a Nanoject II auto-nanoliter injector (Drummond). ZIKV-filled needle is carefully inserted into the thorax of each mosquito and 69 nl of virus (~1000 PFU) is injected. Injected mosquitoes are placed in paper cups with mesh lids and maintained in triple containment for 7 to 10 days. The infection rate of each strain was calculated by comparing infected vs. uninfected mosquitoes (Infection rate (%) = 100 x [# of infected mosquitoes/# of total mosquitoes]) in each experiment.

### Mouse infection experiments

Three to six week old AG129 mice were inoculated with ZIKV via subcutaneous (footpad, a volume of 50 μl) with 10^5^ PFU of ZIKV or anesthetized with Ketamine-Xylazine and fed on by ZIKV infected mosquitoes. Survivals and weight were monitored every day. Mice exhibiting neurologic disease or weight loss of >20% of initial body weight were euthanized. Blood was collected at 1, 3, 5, and 7 dpi in Trizol, RNA was purified and RT-PCR was performed to produce cDNA.

### Viral burden analysis and titration

For mosquitoes, midguts (MG) and salivary glands (SG) were dissected and placed in individual tubes in RLT buffer with 0.1% ß-mercaptoethanol. RNA extraction was performed using RNeasy Mini Kit (QIAGEN) according to manufacturer’s instructions. For mice, chloroform was added to blood samples from ZIKV-infected mice. Tubes were vortexed and centrifuged for 10 min at 14,000 rpm at 4°C. Aqueous layers was mixed with 100% ethanol and RNA was extracted with RNeasy Mini Kit according to manufacturer’s instructions. Extracted RNA was reverse-transcribed with iScript cDNA Synthesis Kit (Bio-rad) according to manufacturer’s protocol. Gene expressions was measured using IQ SYBR Green Supermix. ZIKV RNA levels were normalized to mosquito Rp49 RNA levels or mouse *β* actin RNA levels using the 2^-ΔCt^ calculations. Plaque assays were performed as previously described [23].

### Population genetic analyses

Population structure was evaluated via the Bayesian clustering method implemented by the software STRUCTURE v. 2.3 [24]. The most likely number of clusters (K) was determined by conducting 20 independent runs from each K = 1 to 4. Each run assumed an admixture model and correlated allele frequencies using a burn-in value of 100,000 iterations followed by 500,000 repetitions. The optimal number of K clusters was determined both following the guidelines of [24] and the Delta K method from [21] with the online version of STRUCTURE HARVESTER v. 0.6.94 [24]. Plots of the most biologically informative number of clusters (K = 2) were generated with the program DISTRUCT v.1.1 [25]. Principal Component Analysis (PCA) and Discriminant analysis of Principal Components (DAPC; [26]) were performed on allele frequencies and plotted with the ADEGENET package [27] in R v. 3.2.2. (R Core Team 2013).

### Quantification and statistical analysis

GraphPad Prism software was used to analyze all data. Log_10_ transformed titers used for plaque assays, and either β actin or Rp49 normalized viral RNA or tissue weight-normalized values were analyzed using one-way ANOVA and *post-hoc* Tukey test for multiple comparisons, where appropriate as indicated in figure legends. A p value of <0.05 was considered statistically significant.

## Results

### Genetic distinctness of mosquito strains

**Fig 1:**
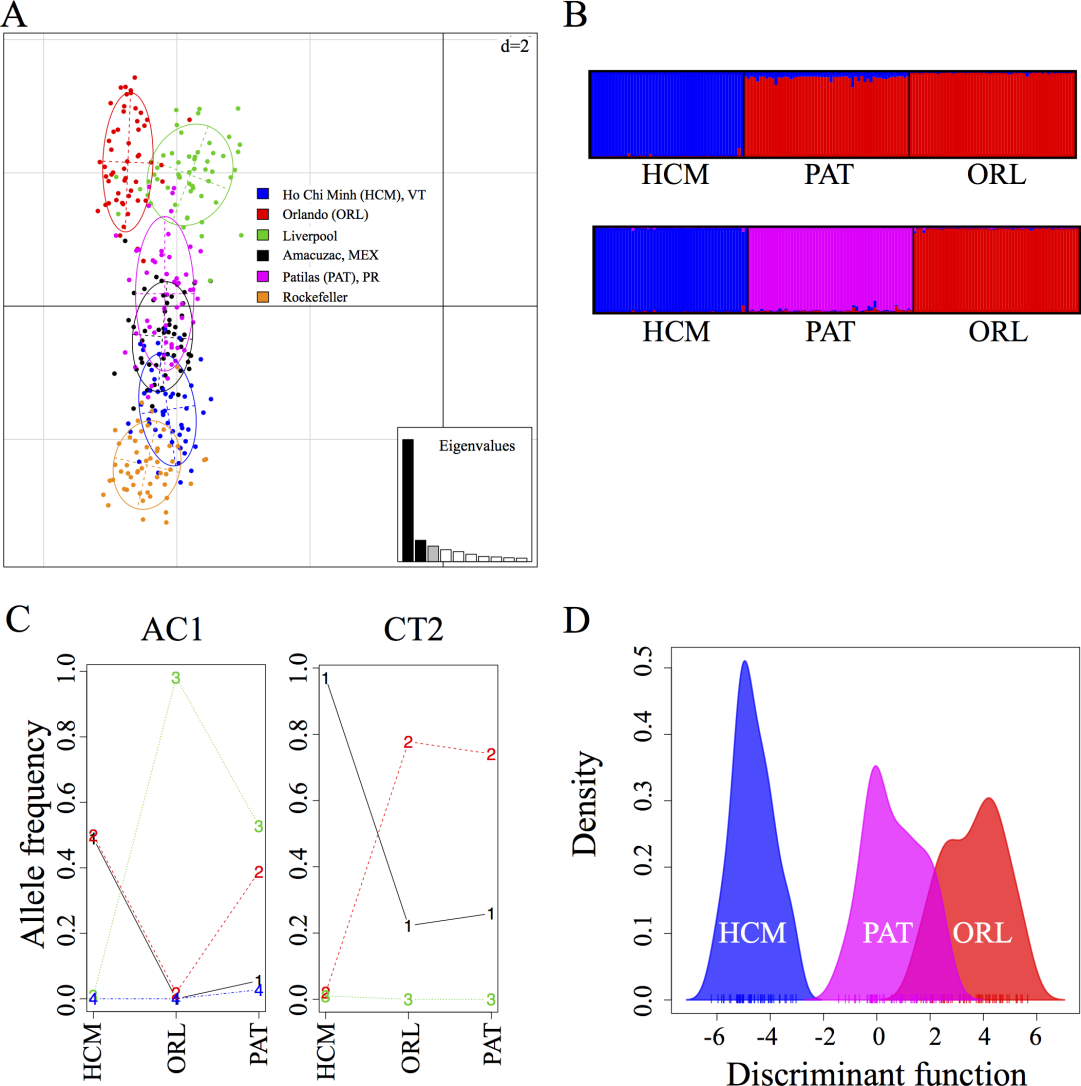
Ho Chi Minh (HCM), Patilas (PAT) and Orlando (ORL) strains of *Aedes aegypti* are genetically diverse. Six strains of *Ae*. *aegypti* (Liverpool, Rockefeller, Ho Chi Minh (HCM), Orlando (ORL), Amacuzac (Mexico) and Patillas (Puerto Rico) strains) were genotyped at 12 microsatellite loci and population genetic analyses were performed to compare these populations. (A) PCA analysis showing the extent of genetic diversity of various laboratory and field-collected strains of *Ae*. *aegypti*. The bar plot with eigenvalues shows the amount of variance represented by each principal component, black bars indicate the components illustrated in these PCA. The units of the grid are indicated at the top right corner. (B) Results of the Bayesian clustering analysis with STRUCTURE [24]. Shown is the bar plot indicating the genetic groupings of the three strains used for ZIKV infections. Each vertical bar represents an individual. The height of each bar represents the probability of assignment to each of (upper panel) K = 2 clusters or (lower panel) K = 3 clusters. Each cluster is indicated by different colours: HCM: blue, PAT: pink and ORL: red. (C) Frequency of alleles highlighting the differences among *Aedes aegypti* laboratory populations from Ho Chi Minh, Orlando Strain, and Patilas, challenged with ZIKV. Two of the 12 microsatellite loci genotyped contributed to the population differentiation observed at a loading threshold of 0.10; AC1 and CT2. More specifically, alleles AC1:209, CT2:184, and CT2:188. Alleles are represented by numbers from 1–4 in the graph for simplification; AC1 (1: 195, 2: 197, 3: 209, 4: 201) and CT2 (1: 188, 2: 184, 3: 196). (D) Discriminant Analysis of Principal Components (DAPC) on microsatellite allele frequencies showing two clear genetic clusters with minimal overlap; colours are as in the upper panel of B.

Genetic strains of *Ae*. *aegypti* vary in their ability to become infected and transmit flaviviruses [11, 28]. We genotyped six representative laboratory and field-derived *Ae*. *aegypti* colonies using 12 microsatellite markers, as described previously [16], and used multiple population genetic analyses to compare them. Principle component analysis (PCA) (Fig 1A) shows a large amount of genetic diversity among all strains. Since it is possible that these genetic differences lead to differences in the capacity for these strains to harbor and transmit ZIKV, we examined three disparate strains in an experimental murine model of mosquito-borne ZIKV infection, a well-studied laboratory strain originally from Florida (Orlando, ORL) and two recently collected strains, one from Vietnam (Ho Chi Minh, HCM) which is shown to be genetically distinct from the ORL strain by PCA analysis and one from Puerto Rico (Patilas, PAT) which can be seen to group between ORL and HCM genetically by PCA analysis (Fig 1A). By varying the optimal number of gene clusters in bayesian clustering analysis [24] from K = 2 (Fig 1B upper panel) to K = 3 (Fig 1B lower panel), we are able to demonstrate that the PAT strain is distinct from both the HCM and the ORL strain, but is closer genetically to the ORL strain. The alleles driving the differentiation among these strains are AC1 and CT2, and the allelic frequencies among the three strains further demonstrate the genetic uniqueness of these mosquito colonies (Fig 1C). Furthermore, a multivariate method of discrimination analysis of principal components–DAPC [27] (Fig 1D) shows that these three strains are genetically distinct, but the PAT strain is more similar to the ORL strain than the HCM strain. A second PCA analysis, using only the three tested strains, also demonstrates the genetic distinction among these groups and suggests a closer genetic similarity between the PAT and ORL strains (S1 Fig).

### Intrathoracic injection of ZIKV into *Ae*. *aegypti*

**Fig 2:**
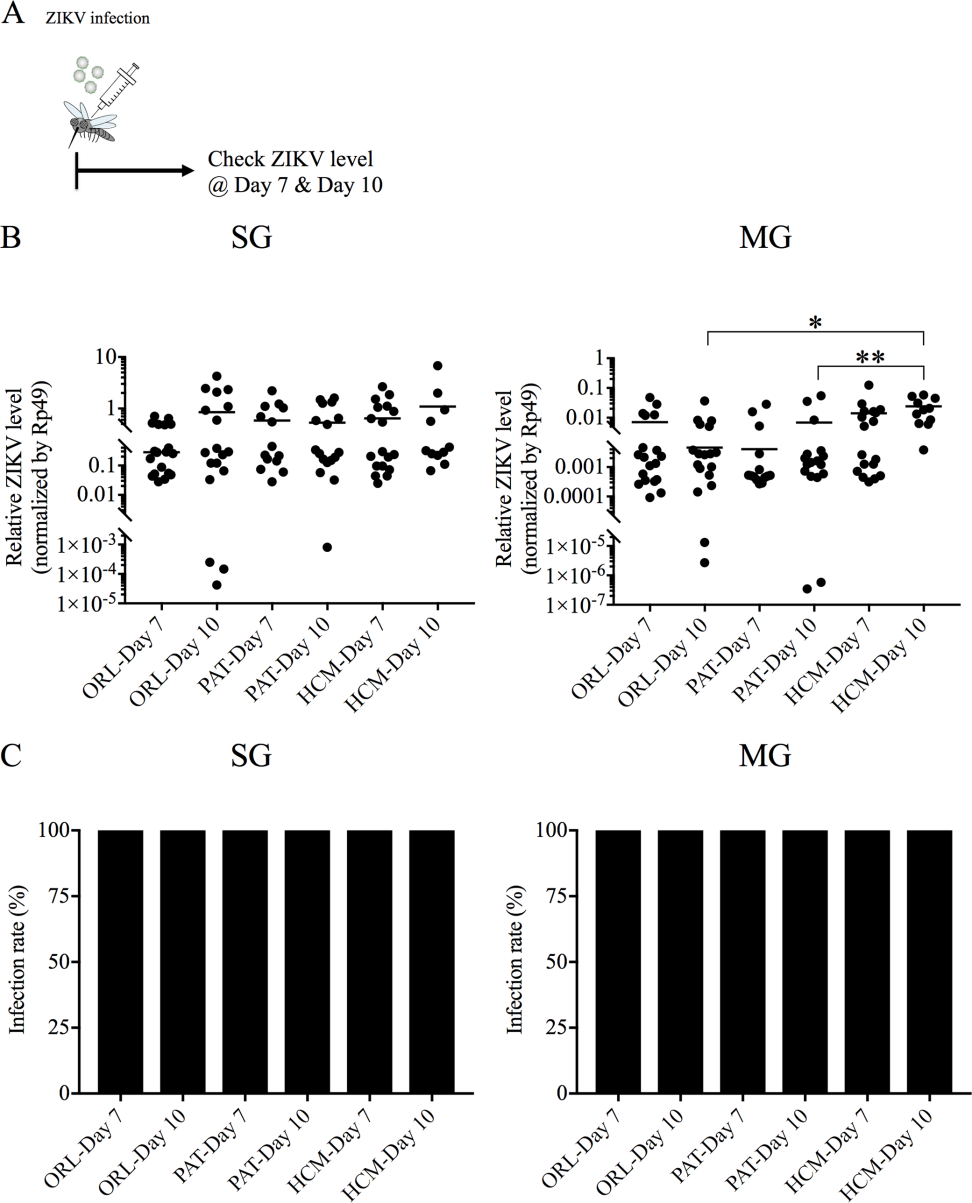
Intrathoracic injection of the Ho Chi Minh (HCM), Patilas (PAT) and Orlando (ORL) strains of *Ae*. *aegypti* with ZIKV results in robust infection of mosquitoes. HCM, PAT and ORL strains of *Ae*. *aegypti* were infected by intrathoracic injection with 10^3^ PFU of ZIKV, and after 7 or 10 days allowed to feed on naïve mice. (A) Experimental workflow for the ZIKV injection experiments. (B) QRT-PCR analysis was performed on the salivary gland (SG) and midgut (MG) of ORL, PAT and HCM mosquitoes 7 or 10 days after injection with ZIKV. ZIKV RNA levels were normalized to mosquito *Rp49* RNA levels. (C) Infection rate of each strain infected for 7 and 10 days was calculated by comparing infected vs. uninfected mosquitoes in each experiment. Data shown are pooled from at least two independent experiments. (n = 19/group for ORL-Day 7; n = 18/group for ORL-Day 10; n = 14/group for PAT-Day 7; n = 17/group for PAT-Day 10; n = 18/group for HCM-Day 7; n = 11/group for HCM-Day 10.) Significance was calculated using one-way ANOVA.

Intrathoracic injection of virus has been shown to generate virus replication in mosquito models for many viruses [29, 30] and, recently, also for ZIKV. To determine if this was also true for our three strains, we injected 10^3^ PFU of ZIKV^Mex^, directly into the thorax of ORL, PAT and HCM strains as described in [31] (Fig 2A). ZIKV replication was detected in SG and MG of all strains of mosquitoes (Fig 2B), and 100% of injected mosquitoes exhibited viral replication in both SG and MG (Fig 2C). In these strains, ZIKV replication was observed in the SG and the MG at 7 and 10 days after inoculation of these mosquitoes (Fig 2B). Interestingly, we observed significantly higher ZIKV levels in the MG of the HCM strain at day 10 as compared to either the PAT or ORL strain, although this experiment uses an artificial infection technique of intrathoracic injection that may not be reflected upon natural infection by blood feeding. These data demonstrate that all tested mosquito strains can sustain ZIKV replication, and the significantly higher infection in the MG of HCM mosquitoes at day 10 after ZIKV injection suggests an increased ability to replicate in these mosquitoes.

### ZIKV transmission after intrathoracic injection

**Fig 3:**
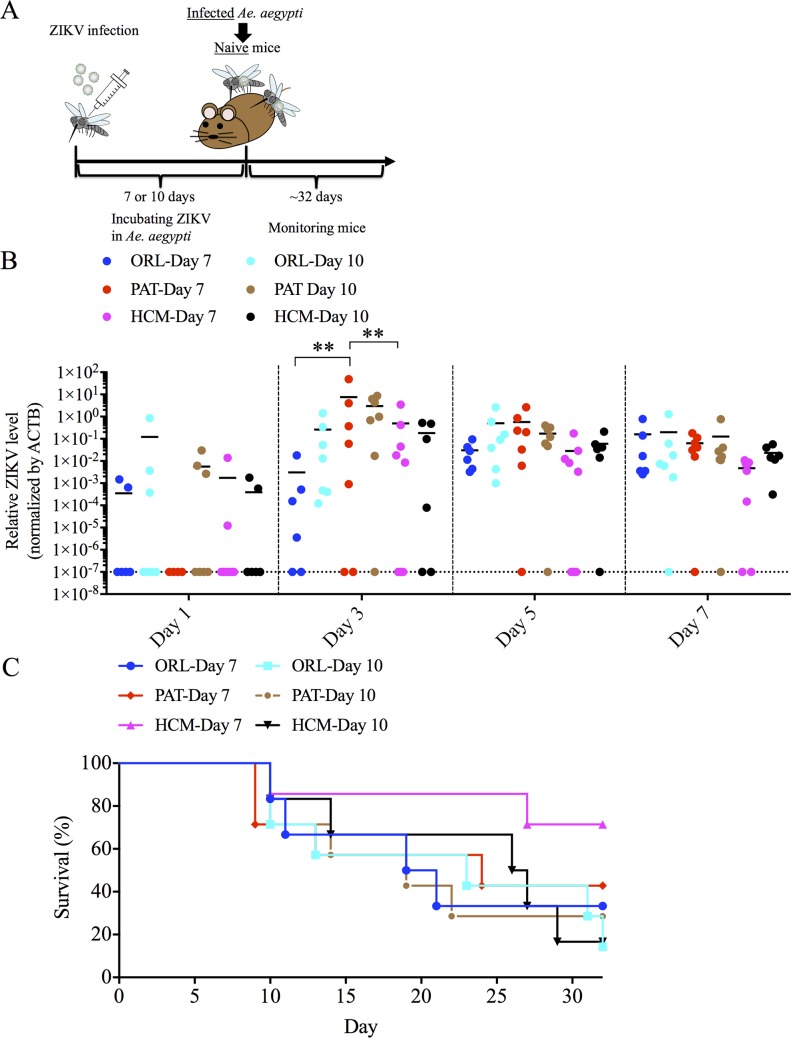
Intrathoracic injection of the Ho Chi Minh (HCM), Patilas (PAT) and Orlando (ORL) strains of *Ae*. *aegypti* with ZIKV results in transmission to mice. HCM, PAT and ORL strains of *Ae*. *aegypti* were infected by intrathoracic injection with 10^3^ PFU of ZIKV, and after 7 or 10 days allowed to feed on naïve mice. (A) Experimental workflow for the ZIKV injection experiments. (B) Blood was collected every other day for one week from naïve mice fed on by mosquitoes intrathoracically-infected with ZIKV for 7 or 10 days, and analyzed for ZIKV infection by qRT-PCR. ZIKV RNA levels were normalized to mouse *β* actin (ACTB) RNA levels. (C) Naïve mice fed on by mosquitoes intrathoracically-infected with ZIKV were monitored for survival for at least 32 days after infected mosquitoes-feeding. Data shown are pooled from at least two independent experiments. (n = 6/group for ORL-Day 7; n = 7/group for ORL-Day 10; n = 7/group for PAT-Day 7; n = 7/group for PAT-Day 10; n = 7/group for HCM-Day 7; n = 6/group for HCM-Day 10.) Significance was calculated using two-way ANOVA with a post-hoc Tukey test. ** P<0.01.

To determine if intrathoracic ZIKV injected mosquitoes could efficiently transmit virus to mice, we fed these infected mosquitoes on naïve mice (Fig 3A). Most mice showed ZIKV RNA in the blood after being fed upon by ZIKV-injected mosquitoes (Fig 3B). Although we observed a significant difference at day 3 post feeding, there was no differences at other tested days after feeding. Of note, 67% (4 of 6 mice, ORL strain, 7 days post injection), 86% (6 of 7 mice, ORL strain, 10 days post injection), 57% (4 of 7 mice, PAT strain, 7 days post injection), 71% (5 of 7 mice, PAT strain, 10 days post injection), 25% (2 of 8 mice, HCM strain, 7 days post injection) and 83% (5 of 6 mice, HCM strain, 10 days post injection) of mice demonstrated significant hind-limb paralysis and were sacrificed after being infected by these mosquitoes, with no significant differences by groups (Fig 3C). To further support that transmitted ZIKV was the cause of death in these mice, we performed plaque assays and showed replicating virus present in the brain of these mice (Table 1). We also examined whether mice that survived virus infection elicited ZIKV-specific antibodies after feeding. We found that most mice with positive ZIKV titers in the serum developed virus-specific antibodies after infected- mosquito feeding, which also supports mosquito-mouse transmission (S1 Table). This mode of transmission is very efficient, as all mosquitoes became infected and could transmit virus, and could be useful for future studies, but we also wanted to demonstrate a more natural route of transmission of ZIKV infection from an infected mammalian host to a mosquito vector and back to a naïve mammalian host. We were also curious if the differences we observed in the higher viral replication in the MG of the HCM strain would be apparent in this mouse-mosquito-mouse cycle.

**Table 1:** Viral titers in brains of mice engorged upon by ZIKV-infected mosquitoes.

Mosquito Strain	Days after infection (Mosquito)	Infection Type (Mosquito)	Day of death (Mouse)	Viral titer in brain (PFU/g)
HCM	10	Oral	11	1.4 x 10^8^
ORL	7	Injection	12	1.0 x 10^8^
ORL	7	Injection	21	5.0 x 10^2^
ORL	10	Injection	10	1.8 x 10^8^
ORL	10	Injection	10	1.8 x 10^8^
ORL	10	Injection	23	2.4 x 10^7^
PAT	7	Injection	9	3.3 x 10^8^
PAT	7	Injection	13	2.1 x 10^8^
PAT	7	Injection	25	6.5 x 10^6^
PAT	10	Injection	10	1.1 x 10^8^
PAT	10	Injection	14	1.6 x 10^7^
HCM	7	Injection	27	3.0 x 10^4^
HCM	10	Injection	14	2.7 x 10^7^
HCM	10	Injection	26	1.0 x 10^6^
HCM	10	Injection	27	9.7 x 10^4^

ORL, PAT or HCM strains were infected with ZIKV, either orally or by intrathoracic injection, for 7 or 10 days before being allowed to take a blood meal on naïve AG129 mice. When mice died, brains were collected, and plaque assays were performed on the samples.

### Infection of *Aedes aegypti* after taking a blood-meal on ZIKV-infected mice

**Fig 4:**
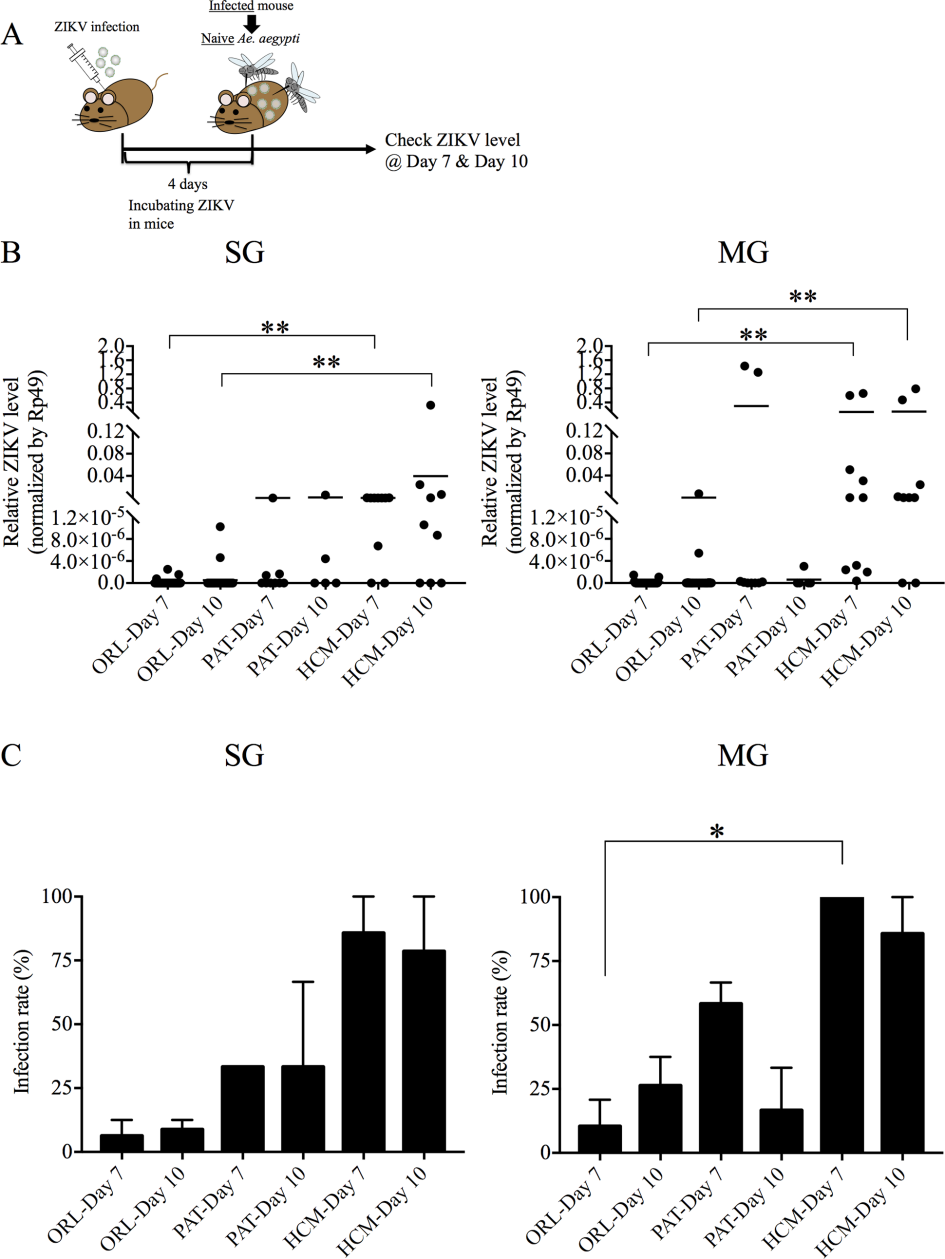
Vector capacity of ZIKV in Ho Chi Minh (HCM), Patilas (PAT) and Orlando (ORL) strains of *Ae*. *aegypti*, after taking a blood meal on mice. HCM, PAT and ORL strains of *Ae*. *aegypti* were infected with ZIKV by allowing mosquitoes to ingest a blood meal from mice infected with ZIKV. (A) Experimental workflow for the ZIKV oral infection experiments. (B) ZIKV levels by qRT-PCR of the salivary glands (SG) and midgut (MG) of ORL, PAT and HCM mosquitoes 7 or 10 days after infection. ZIKV RNA levels were normalized to mosquito *Rp49* RNA levels. (C) Infection rate of each strain infected at 7 and 10 days was calculated by comparing infected vs. uninfected mosquitoes in each experiment. Data shown are pooled from at least two independent experiments. (n = 35/group for ORL-Day 7; n = 28/group for ORL-Day 10; n = 8/group for PAT-Day 7; n = 5/group for PAT-Day 10; n = 10/group for HCM-Day 7; n = 9/group for HCM-Day 10.) Each data point represents an individual mosquito. Significance was calculated using one-way ANOVA. * P<0.05, ** P<0.01.

To examine whether ZIKV can be transmitted from infected mice to *Ae*. *aegypti*, we subcutaneously infected AG129 mice with 10^5^ PFU of ZIKV. Four days after infection, the ORL, PAT and HCM strains of *Ae*. *aegypti* were allowed to ingest a blood meal from the ZIKV-infected mice in which the ZIKV level was 10 ^5.0±0.3^ plaque-forming-units (PFU) equivalents per ml (Fig 4A). ZIKV levels were low or undetectable in MG and SG of the ORL strain at 7 and 10 days after feeding, and the results varied in the PAT strain with some mosquitoes exhibiting higher levels of virus and some showing very low levels (Fig 4B). Higher levels of ZIKV were detected in the MG of the HCM strain as compared to the ORL strain at both time points, and as the PAT strain at day 10 post-feeding (Fig 4B). HCM mosquitoes also showed more virus replication in the SG relative to ORL, while the PAT strain had some mosquitoes that demonstrated virus levels closer to the HCM strain (Fig 4B). Overall, the infection rates were significantly different among the ORL, PAT and HCM strains, with the PAT strain, interestingly, exhibiting a phenotype that is between the HCM and ORL strains but closer to the ORL strain (Fig 4C). This intermediate phenotype demonstrated in the PAT strain could be interpreted as the genetic differences among the strains, demonstrated in Fig 1A–1D, is capable of driving a difference in vector competency, although other differences could also play a role.

### ZIKV transmission after oral feeding

**Fig 5:**
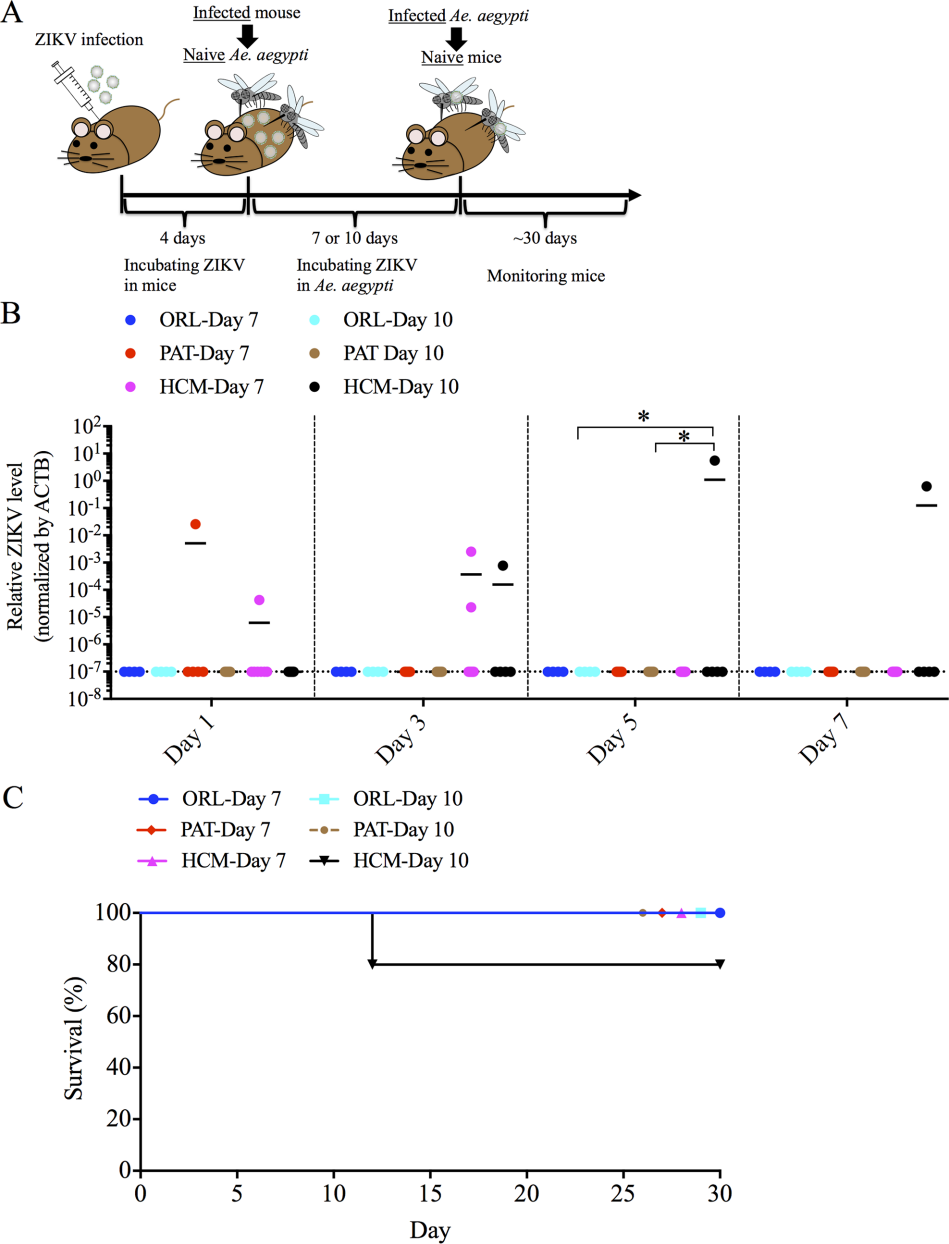
Transmission of ZIKV to naïve mice in Ho Chi Minh (HCM), Patilas (PAT) and Orlando (ORL) strains of *Ae*. *aegypti*, after taking a blood meal on mice. HCM, PAT and ORL strains of *Ae*. *aegypti* were infected with ZIKV by allowing mosquitoes to ingest a blood meal from mice infected with ZIKV. (A) Experimental workflow for the ZIKV oral infection experiments. (B) Blood was collected every other day for one week from naïve mice fed on by mosquitoes infected with ZIKV for 7 or 10 days, and analyzed for ZIKV infection by qRT-PCR. ZIKV RNA levels were normalized to mouse *β* actin (ACTB) RNA levels. (C) Naïve mice fed on by mosquitoes orally-infected with ZIKV were monitored for survival for at least 30 days after infected mosquitoes-feeding. Data shown are pooled from at least two independent experiments. (n = 8/group for ORL-Day 7; n = 8/group for ORL-Day 10; n = 5/group for PAT-Day 7; n = 5/group for PAT-Day 10; n = 7/group for HCM-Day 7; n = 5/group for HCM-Day 10.) Each data point represents an individual mouse. Significance was calculated using two-way ANOVA with a post-hoc Tukey test. * P<0.05.

To assess whether mosquitoes which fed on ZIKV-infected mice are capable of transmitting ZIKV to naïve mice, we allowed these ZIKV-infected mosquitoes to engorge on naïve mice (Fig 5A). No animals fed on by the ZIKV-infected ORL mosquitoes developed viremia or died, consistent with the low or undetectable level of ZIKV in the SG of these mosquitoes (Fig 5B). ZIKV was detected, in the blood fed on by the PAT strain in 1 of 5 mice (PAT strain, 7 days post blood meal), in HCM strain in 2 of 7 mice (HCM strain, 7 days post blood meal) and 1 of 5 mice (HCM strain, 10 days post blood meal), respectively (Fig 5B), but no mice fed upon by ORL strain mice (0 of 8 mice on day 7 and 0 of 8 on day 10) or by PAT strain at day 10 of mosquito infection (0 of 5 mice). In addition, the mouse on which the most highly infected HCM mosquito (Fig 4B) fed developed the ZIKV viremia (Fig 5B), and by day 12 this mouse developed severe paralysis and was sacrificed due to ZIKV infection, which was confirmed by a replicating virus present in the brain (Fig 5C, Table 1). These results show that ZIKV can migrate from infected mice to *Ae*. *aegypti*, disseminate from the MG to the SG, and then be transferred back to naïve mice when mosquitoes ingest a blood meal. The transmission rate however, appears to be very low and depends on the ability for the *Ae*. *aegypti* strain to replicate in both the MG and the SG.

A previous study demonstrated that the bacterial microbiome can alter mosquito vector competency [32]. These strains were all raised in the same environment for at least 3 generations, suggesting that the overall composition of the microbiome should be similar, but there is a chance that the ability for control of bacteria in the MG varies by strain. Therefore, to determine if these mosquitoes harbor significantly different levels of bacteria in their MG, an analysis of the overall 16s bacteria load was performed and no differences were observed (S2 Fig).

## Discussion

*Ae*. *aegypti* is one of the most genetically diverse species of insect ever studied, and has the ability to rapidly adapt to human habitats [16]. Here, we established an *Ae*. *aegypti* mosquito model of ZIKV infection of mice. Consistent with recent studies, ZIKV transmits from infected mosquitoes to mice [14, 15, 33]. This study used AG129 mice for our transmission model, and this model has advantages for analyzing the pathogenesis of ZIKV or the effect of mosquito factors on ZIKV transmission and infectivity. We also demonstrated variability in the susceptibility of three genetically diverse colonies of *Ae*. *aegypti*, the ORL, PAT and HCM strains, to ZIKV infection. As viral replication in SG is important for ZIKV transmission to the vertebrate host, our results suggest that the HCM strain may be more capable than the ORL or PAT strain of transmitting virus. Also, these results were consistent with recent studies that suggest dissemination of ZIKV in *Ae*. *aegypti* depends on both virus and mosquito strains [17, 34]. These observations have implications for the further spread of ZIKV into distinct populations of mosquitoes across the globe. There are multiple mechanisms by which the difference in vector capacity among strains could arise, such as genetic variation in metabolic and/or immune pathways, or entry or replication factors necessary for ZIKV infection [35]. While it is impossible to make any definitive statements on genes that drive differences in vector competency based solely on the location of the microsatellite markers used to analyze genetic variability, we are able to indicate genes that are proximal to the markers driving variability among the groups, particularly AC1 and CT2 (Table 2). In order to make a more definitive statement on genes involved in vector competency, further experiments crossing the strains and mapping single nucleotide polymorphisms (SNPs) or targeted gene modification is necessary.

**Table 2:** Genes proximal to microsatellite clusters driving genetic variability in mosquito strains.

Microsatellite	Gene Name	Putative Function (If known)
AC1	AAEL011788	Ankyrin-like protein with endonuclease activity
AC1	AAEL011789	Probable citrate synthase 2, mitochondrial precursor
AC1	AAEL011790	ESCRT-II complex protein
AC1	AAEL011791	Uncharacterized protein with multiple coiled coils
AC1	AAEL011792	Putative potassium/chloride symporter
AC1	AAEL011793	Putative aspartyl beta-hydroxylase
AC1	AAEL011794	Uncharacterized protein with F-box domain
CT2	AAEL004149	Uncharacterized transmembrane protein
CT2	AAEL004155	Ovary ecdysteroidogenic hormone I
CT2	AAEL004159	T-box transcription factor tbx6
CT2	AAEL004163	Putative O-fucosyltransferase
CT2	AAEL004164	Uncharacterized protein
CT2	AAEL004169	Uncharacterized protein
CT2	AAEL004174	T-box transcription factor tbx6
CT2	AAEL004179	Putative ion channel
CT2	AAEL004180	Ubiquinone biosynthesis monooxygenase
CT2	AAEL004181	Putative tRNA (Glu)-specific nuclease WapA
CT2	AAEL004193	Putative rophilin protein with PDZ domain
CT2	AAEL004195	Membrane associated progesterone receptor
CT2	AAEL004197	Uncharacterized protein

Two microsatellite markers were used for the genetic analysis in Fig 1. They are termed **AC1**, located at AAGE02021534.1; supercont1.679, and **CT2**, located at AAGE02006436.1; supercont1.109. Genes within 0.5 Mb upstream and downstream of these microsatellites were searched using VectorBase to identify putative protein functions.

After a mosquito ingests an infectious blood meal, several steps are required for viral infection and subsequent transmission back to a mammalian host. Two types of MG and SG barriers are known to prevent infection by other viruses: one barrier prevents or controls virus infection of epithelial cells in the MG or SG, and the other is a MG or SG escape barrier which can block virus from escaping from the MG epithelial cells into the hemolymph or from the SG epithelial cells into the lumen of the SG [35, 36]. Since ZIKV levels were very similar among the three strains during intrathoracic injection but the ORL and PAT strains were lower during oral feeding, it seems that the ORL and PAT strains harbor a MG barrier to infection related to either the ability to replicate ZIKV in the MG or for the virus to escape once it does replicate.

This barrier appears to be related to the underlying genetic differences among the strains, but it is also possible that the microbiome composition of the mosquito MG contributes to these phenotypes [37]. The vast majority of the microbiome of the mosquito is acquired from the environment in which it was raised [38], and we did not observe a difference in total bacteria burden. This further strengthens our hypothesis that the differences observed in vector competence are directly related to the genetic differences demonstrated in Fig 1, but clearly the observed phenotypic differences could be more complex and the composition of the microbiome, or even the insect-specific viriome have the potential to play a role in these infections.

Alternatively, previous studies on related viruses have demonstrated that the bacterial microbiome is capable of modulating mosquito vector competence [32]. It has been suggested that bacteria affect arbovirus replication in mosquitoes by regulating immunity, affecting the production of metabolites, and inducing anti-viral miRNA expressions [32, 39, 40]. Our data indicate that the overall microbiome is unchanged among these strains.

During transmission from mosquitoes to mammalian hosts, SG proteins from the flaviviral vector, *Ae*. *aegypti*, are known to enhance viral replication and pathogenesis of both dengue and West Nile virus in mammalian hosts [41–43]. It is also clear that the immune response to specific vector proteins in the saliva of mosquitoes can alter the severity of flaviviral disease for individual patients [44]. These findings raise the possibility that specific *Ae*. *aegypti* SG components can facilitate greater ZIKV infection *in vivo*. The ZIKV mosquito to mouse transmission model established here is crucial for the exploration into this phenomenon for this important human pathogen. Many studies into ZIKV have been performed using needle inoculation of virus into a mouse model [19, 45, 46], an unnatural route of infection that does not recapitulate what occurs in nature. While the data gained from these studies have undoubtedly uncovered interesting mechanisms of ZIKV replication and pathogenesis, the development here of a mouse-to-mosquito-to-mouse transmission model representing a natural route of infection has the potential to reveal aspects of the viral life cycle that have heretofore remained undiscovered. In addition, further investigation into the mechanisms that underlie the differences in vector capacities of these three strains has the potential to elucidate aspects of the life cycle of ZIKV in the *Ae*. *aegypti* mosquito.

## Supporting information

S1 Fig The genetic diversity of Ho Chi Minh (HCM), Patilas (PAT) and Orlando (ORL) strains of *Aedes aegypti* related to Fig 1A. PCA analysis showing the extent of genetic diversity of various laboratory and field-collected strains of *Ae*. *aegypti*, including Ho Chi Minh (HCM), Orlando (ORL) and Patillas (PAT) strains that are used in this study. The bar plot with eigenvalues shows the amount of variance represented by each principal component, black bars indicate the components illustrated in these PCA. The units of the grid are indicated at the top right corner.
(TIFF)

S2 Fig No difference of total bacterial burden among Ho Chi Minh (HCM), Patilas (PAT) and Orlando (ORL) strains of *Aedes aegypti* after intrathoracic injection or oral feeding. The microbial load in the MG was determined 10 days after intrathoracic injection (A) or oral feeding (B) by qRT-PCR. Total bacterial 16S rRNA levels were normalized to mosquito *Rp49* RNA levels. One dot represents one mosquito gut. The horizontal line represents the median of the results. The results were combined from at least two biologically independent experiments.
(TIFF)

S1 Table Antibody responses of ZIKV-replicated AG129 mice engorged upon by ZIKV-infected mosquitoes. ORL, PAT or HCM strains were infected with ZIKV, either orally or by intrathoracic injection, for seven or ten days before being allowed to take a blood-meal on naïve AG129 mice. After 4–5 weeks after feeding, serum of mice in which ZIKV were detected in blood were collected and antibody responses were analyzed by ELISA.
(PDF)
