# Supplementary figures and images for: Altered vector competence in an experimental mosquito-mouse transmission model of Zika infection
Source: PLoS Negl Trop Dis. 2018 Mar 5;12(3):e0006350. doi: 10.1371/journal.pntd.0006350 (PMC5854422; doi:10.1371/journal.pntd.0006350)

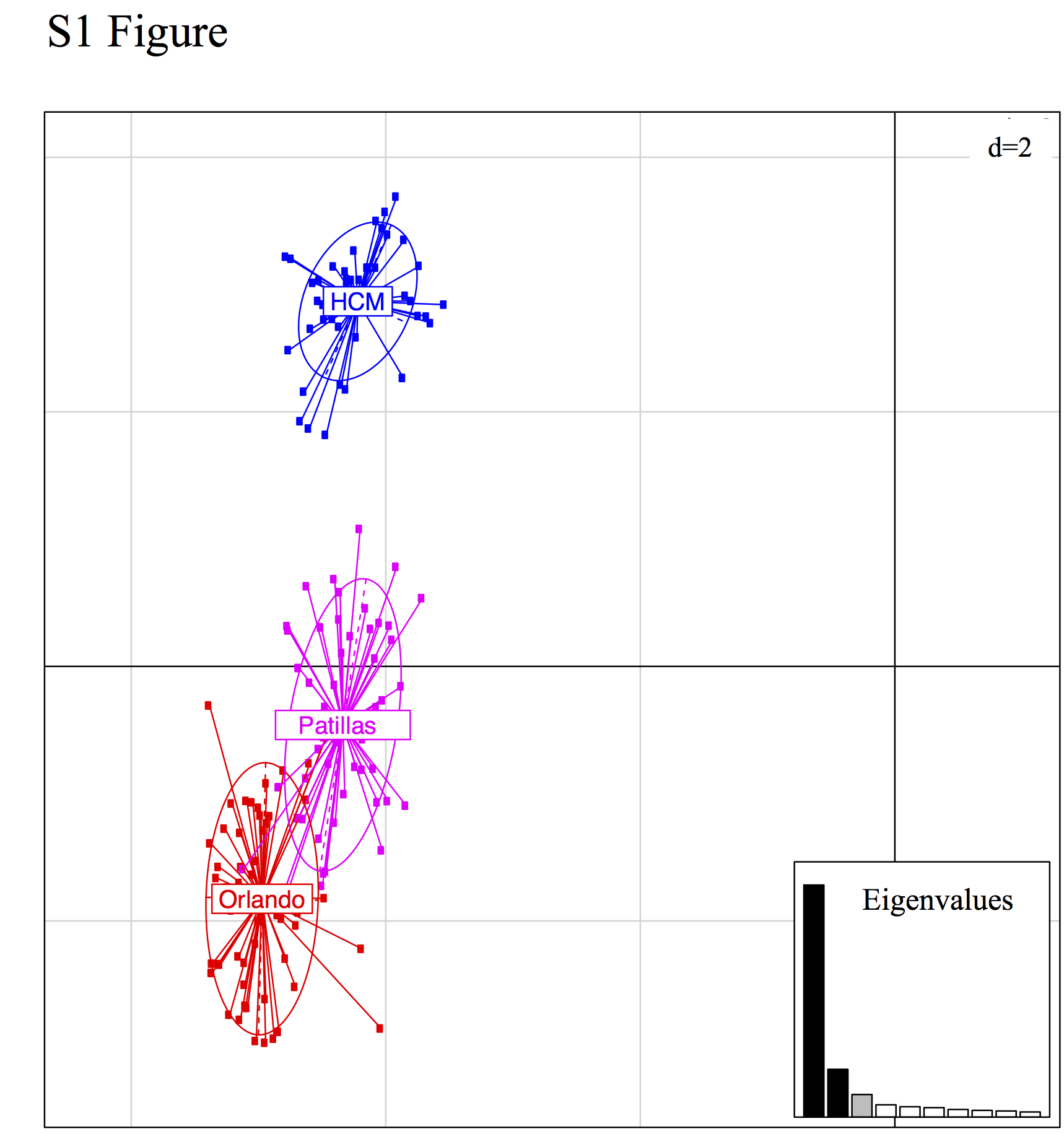

Supplement: S1 Fig — PCA analysis showing the extent of genetic diversity of various laboratory and field-collected strains of Ae. aegypti, including Ho Chi Minh (HCM), Orlando (ORL) and Patillas (PAT) strains that are used in this study. The bar plot with eigenvalues shows the amount of variance represented by each principal component, black bars indicate the components illustrated in these PCA. The units of the grid are indicated at the top right corner. (TIFF) [file pntd.0006350.s001.tiff]

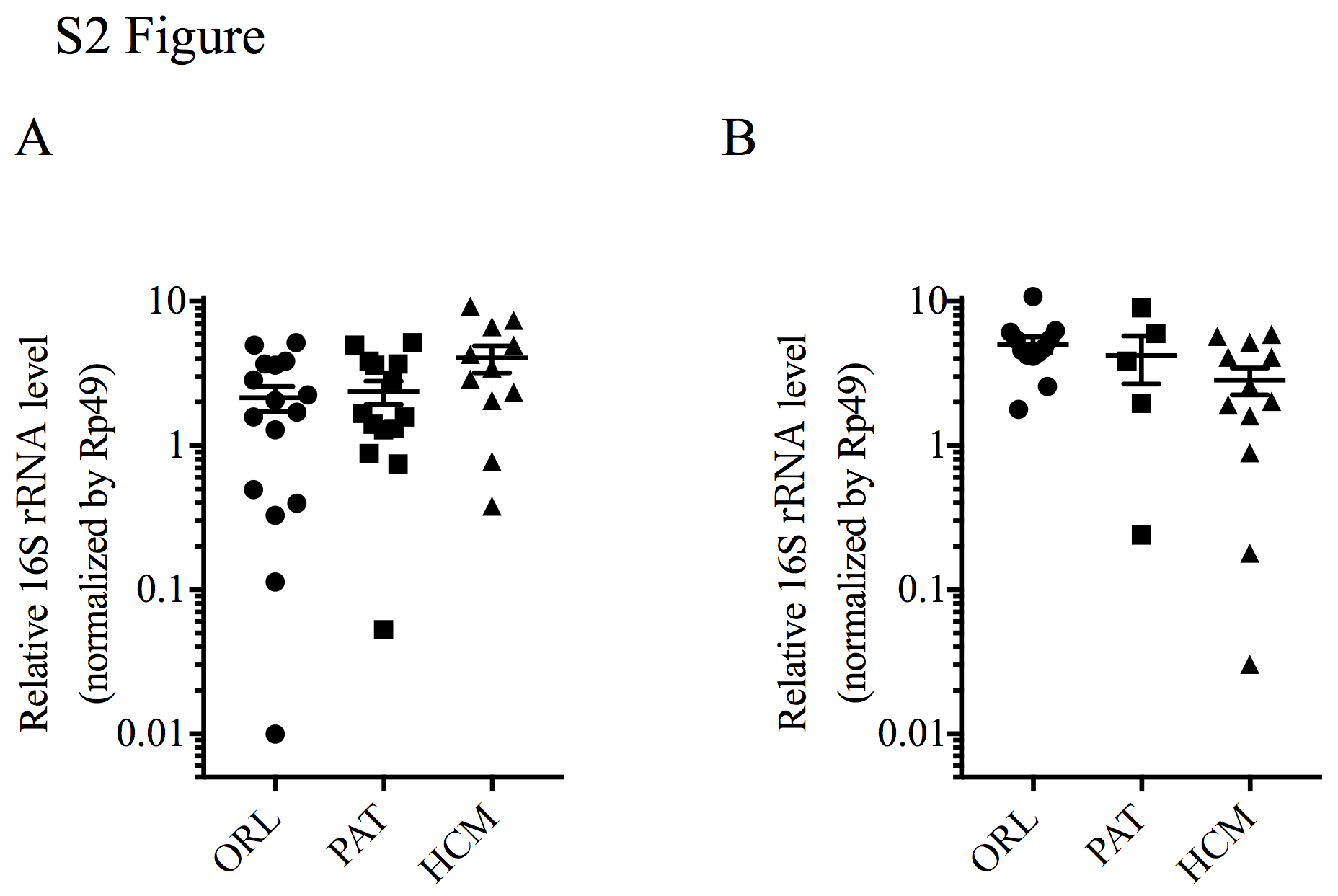

Supplement: S2 Fig — The microbial load in the MG was determined 10 days after intrathoracic injection (A) or oral feeding (B) by qRT-PCR. Total bacterial 16S rRNA levels were normalized to mosquito Rp49 RNA levels. One dot represents one mosquito gut. The horizontal line represents the median of the results. The results were combined from at least two biologically independent experiments. (TIFF) [file pntd.0006350.s002.tiff]
