# Supplementary material for: Altered vector competence in an experimental mosquito-mouse transmission model of Zika infection
Source: PLoS Negl Trop Dis. 2018 Mar 5;12(3):e0006350. doi: 10.1371/journal.pntd.0006350 (PMC5854422; doi:10.1371/journal.pntd.0006350)
Supplement: S1 Table — ORL, PAT or HCM strains were infected with ZIKV, either orally or by intrathoracic injection, for seven or ten days before being allowed to take a blood-meal on naïve AG129 mice. After 4–5 weeks after feeding, serum of mice in which ZIKV were detected in blood were collected and antibody responses were analyzed by ELISA. (PDF) [file pntd.0006350.s003.pdf]

Supplementary Table 1. Antibody responses of ZIKV-replicated AG129 mice engorged upon by ZIKV-infected mosquitoes.

Mosquito Strain	Days after infection (Mosquito)	Infection Type (Mosquito)	Antibody titers
PAT	7	Oral	<20
HCM	7	Oral	<20
HCM	7	Oral	<20
ORL	7	Injection	640
ORL	10	Injection	40
PAT	7	Injection	160
PAT	7	Injection	1280
PAT	10	Injection	640
HCM	7	Injection	<20
HCM	7	Injection	320
HCM	7	Injection	320
HCM	7	Injection	80
HCM	10	Injection	<20
